# Uncommon Etiology of Pancreatic Mass: a Case Report

**DOI:** 10.7759/cureus.66879

**Published:** 2024-08-14

**Authors:** Sanae Moqran, Layla Tahiri El Ousrouti, Nawal Hammas, Nizar El Bouardi, Laila Chbani

**Affiliations:** 1 Laboratory Medicine, Faculty of Medicine, Pharmacy and Dental Medicine, Fez, MAR; 2 Pathology, Hassan II University Hospital, Fez, MAR; 3 Radiology, Hassan II University Hospital, Fez, MAR

**Keywords:** aip, autoimmune, pancreatic cancer, pancreatitis, igg4 related disease

## Abstract

IgG4-related autoimmune pancreatitis (AIP) is a chronic inflammatory disease of the pancreas with a distinct histological feature. Its diagnosis remains challenging as some features overlap with pancreatic cancer. We present a case of IgG4-related AIP mimicking pancreatic cancer. A 70-year-old male patient presented with epigastric pain, radiating to the entire abdomen with an unquantified weight loss. Magnetic resonance cholangiopancreatography (MRCP) showed a mass with a 28 mm long axis, in the head of the pancreas with pancreatic duct dilatation. Thus, it was presumed to be a pancreatic neoplasm and pancreatic resection was undertaken without a definitive preoperative diagnosis. In terms of clinical presentation, imaging characteristics, and laboratory parameters, IgG4-related AIP can resemble pancreatic cancer. Thus, histopathological studies remain the gold standard for a definitive diagnosis that may show a diffuse lymphoplasmacytic infiltrate with storiform fibrosis. On immunohistochemistry, the majority of plasma cells are positive for IgG4 (>50 per high-power field (HPF)). In our case, the histologic diagnosis allowed us to suggest the diagnosis of IgG4-related AIP and the immunohistochemical diagnosis confirmed the diagnosis. It is critical to distinguish pancreatic cancer from IgG4-related AIP due to its completely different prognosis and therapy. Steroids are the first-line treatment that allow a reduction of risk of relapse; therefore, a misdiagnosis as a malignancy leads to inappropriate surgical interventions. In this case, a biopsy is recommended.

## Introduction

Autoimmune pancreatitis (AIP) is an uncommon form of chronic pancreatitis caused by an immunological reaction, resulting in inflammation in the pancreas. Two types of AIP exist: type 1 or IgG4-related AIP, and type 2, which is also known as idiopathic duct-centric pancreatitis, a disease unrelated to IgG4. Type 1 AIP is characterized by pancreatic symptoms that occur within a broader systemic fibroinflammatory disorder, known as IgG4-associated, which can affect various organs in the body [1].

It should be noted that there are overlapping clinical and radiological symptoms between the two conditions. A precise diagnosis requires a combination of clinical, radiological, serological, and histopathological examination. The main differential diagnosis is pancreatic cancer, underscoring the paramount need for an accurate diagnosis. Accurate diagnosis is crucial as steroid medication has been demonstrated to be highly effective, and surgical intervention is redundant in most cases.

This article was previously presented as a poster at the 35th European Congress of Pathology in September 2023 and subsequently published as an abstract in the Virshow archive.

## Case presentation

**Figure 1:**
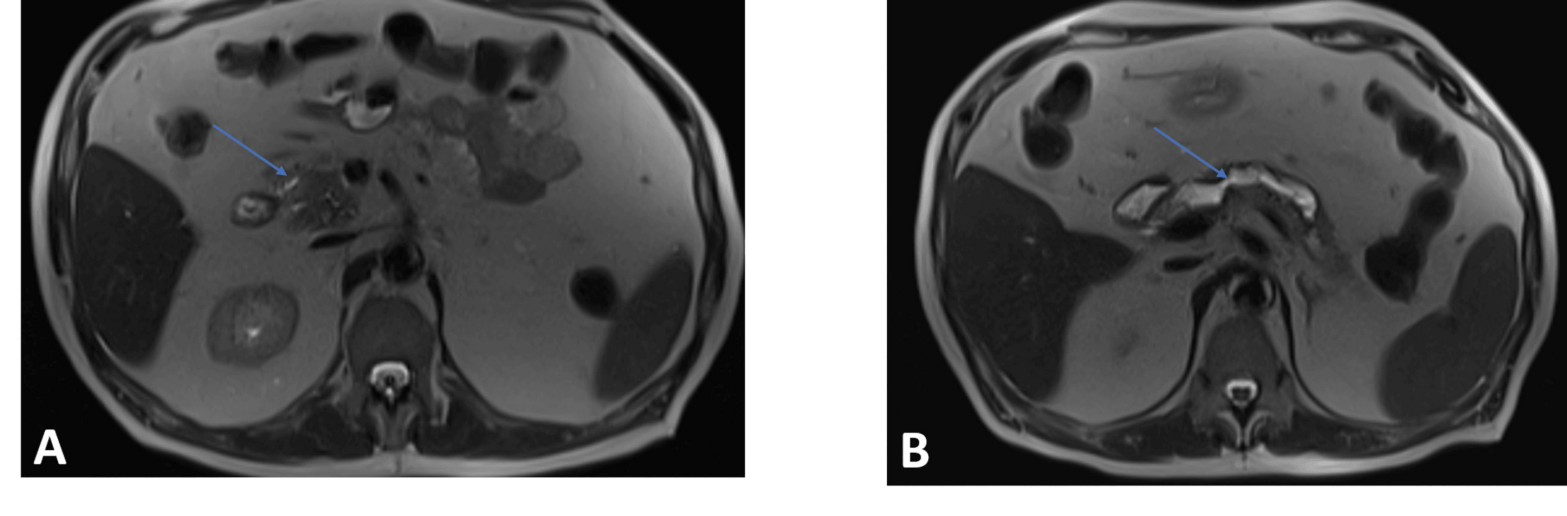
Presence of a cephalic pancreatitis mass (A) resulting in the dilation of the upstream Wirsung duct (B)

A 70-year-old male patient, who was undergoing hemodialysis for end-stage renal failure and had a chronic interstitial lung disease associated with fibrosis, presented with epigastric pain radiating to his whole abdomen and unquantified weight loss. No jaundice was observed. The patient's test results lacked specificity, with bilirubin at 4.6, glutamic oxaloacetic transaminase (GOT)/glutamic pyruvic transaminase (GPT) at 25/11.3, alkaline phosphatase (ALP)/gamma-glutamyl transferase (GGT) at 80/77, and tumour marker values of CA-19 at 65.6 U/mL (reference range, 0-35 U/mL). Magnetic resonance cholangiopancreatography (MRCP) revealed a 28 mm mass in the pancreatic head, causing upstream main pancreatic duct dilatation (Figure 1).

**Figure 2:**
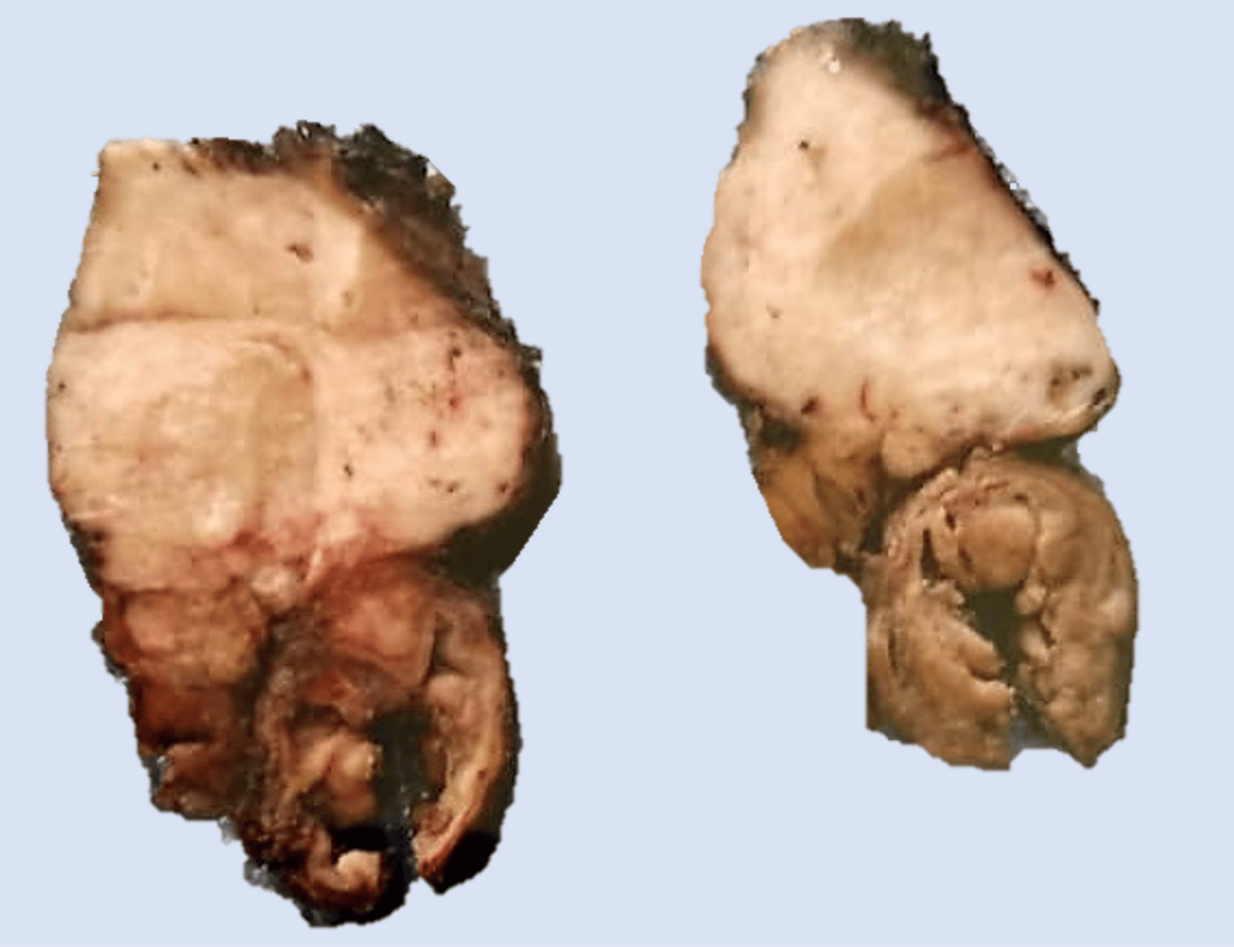
Gross evaluation showing a firm and white unlimited mass in the head of pancreas

Based on these findings, the presence of a pancreatic neoplasm was suspected, and the patient proceeded to undergo pancreatic resection without a clear preoperative diagnosis. On gross evaluation (Figure 2), a solid and white mass was found in the head of the pancreas.

**Figure 3:**
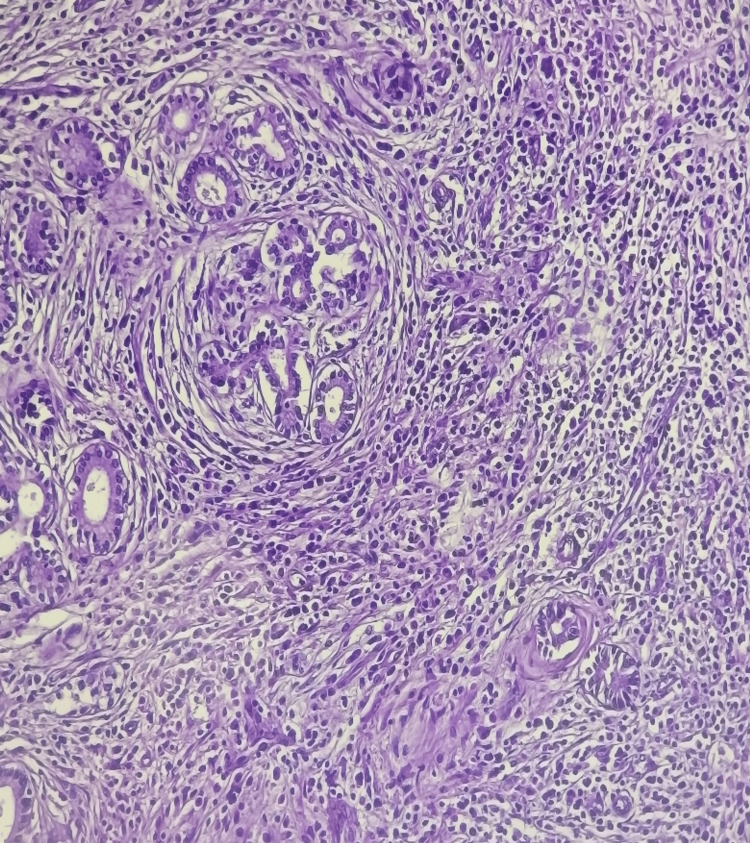
Diffuse lymphoplasmacytic infiltration of the pancreas and marked storiform fibrosis.

**Figure 4:**
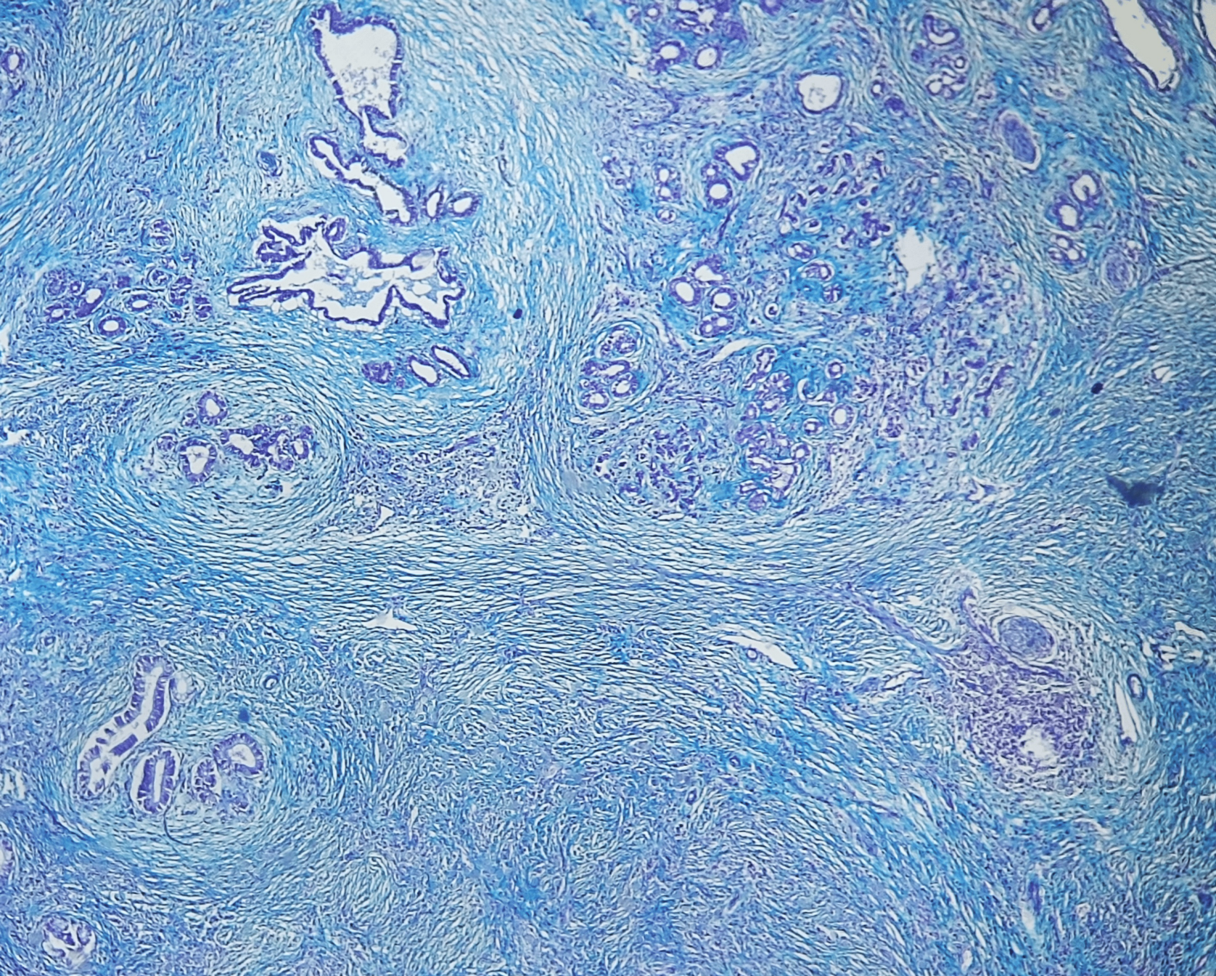
Storiform fibrosis more discernible after Masson’s trichrome staining.

On histological examination, it was revealed that the pancreatic parenchyma was largely separated by storiform collagen bundles and accompanied by a dense, inflammatory infiltrate that contained a high number of plasma cells (Figures 3, 4).

**Figure 5:**
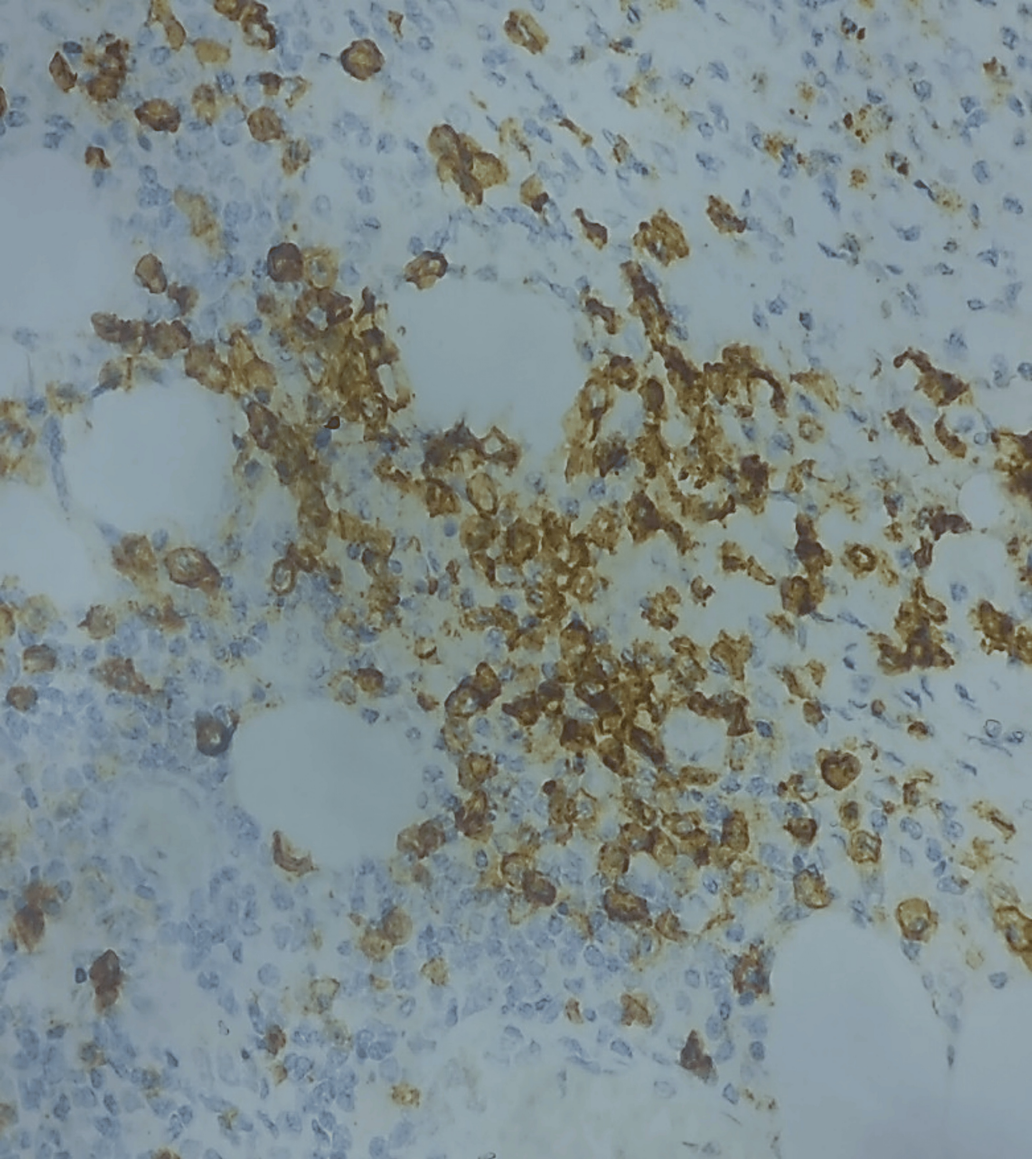
Highly magnified view showing diffuse infiltration by CD138-positive plasma cells.

**Figure 6:**
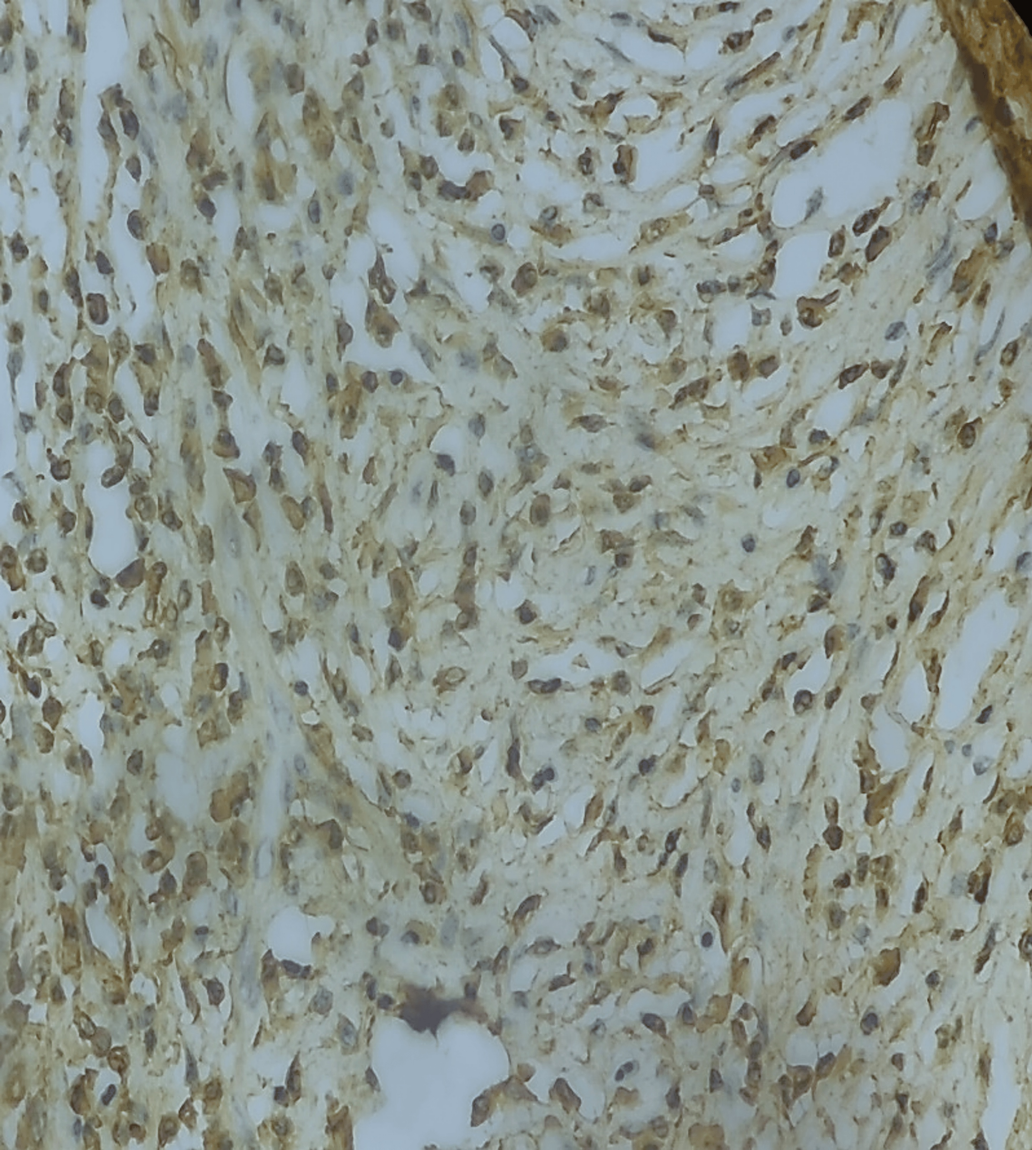
IgG4-positive plasma cells seen in abundance in the pancreas.

Based on the results, AIP IgG4-related diagnosis was suspected and additional immunohistochemical tests were required to confirm the diagnosis. On immunohistochemistry, the anti-CD138 antibody verified the prevalence of plasma cells, most of which were positive for IgG4 (Figures 5, 6).

## Discussion

IgG4-related AIP represents the pancreatic manifestation of IgG4-associated disease, which is a systemic fibroinflammatory disorder. The first report of this condition dates back to 1892, when Muckilicz described a patient with symmetrical enlargement of salivary glands and extensive mononuclear cell infiltration [1]. In 1991, Kawaguchi identified the histological features of pancreatic AIP, naming it lymphoplasmacytic sclerosing pancreatitis (LPSP), four years prior to Yoshida's proposal of the term PAI [2]. It took Hamano another decade to discover the association between elevated IgG4 serum levels and LPSP [3]. Studies subsequent to this publication provided evidence of the infiltration of inflammatory tissues with plasma cells containing IgG4 antibodies in the pancreas of LPSP patients [4]. In 2003, a Japanese team reported the phrase 'IgG4-related AIP' as a novel clinicopathological entity within the wider multifocal disorder of IgG4-associated diseases [5].

Due to the novelty of this diagnosis and lack of recognition, there is limited understanding of the epidemiology of this disease. Japanese researchers showed great interest in studying this recently discovered entity. The Japanese National Epidemiological Survey indicated a prevalence of 4.6/100000 individuals and an incidence of 1.4 per 100,000. It is more probable to affect men than women. The Japanese survey reported a mean patient age of 68.1 years [6], reminiscent of pancreatic cancer's demographic profile.

IgG4-related AIP presents commonly with painless obstructive jaundice resulting from biliary obstruction, in addition to non-specific symptoms such as nausea, vomiting or weight loss [7]. Laboratory tests are not specific and only elevated serum IgG4 levels have been described in numerous series, with sensitivity and specificity ranging from 67% to 94% and 89% to 100%, respectively, according to a meta-analysis of seven studies [8].

Abdominal computed tomography (CT) and magnetic resonance imaging (MRI) are frequently employed to assess alterations in the pancreatic parenchyma. In the context of AIP, these imaging techniques demonstrate either diffuse hypertrophy or focal hypertrophy of the pancreas, contingent on the extent of the pathology. In cases of focal hypertrophy, pancreatic cancer should be considered as a primary diagnosis in order to exclude it. In the presented case, AIP and pancreatic cancer share numerous clinical and radiological features, including a pancreatic mass and dilatation of the main upstream pancreatic duct.

Upon gross evaluation, the disease predominantly affects the pancreatic head. On occasion, the entire gland is affected as well. The pancreas exhibits a firm to hard consistency. The presence of a discernible mass is infrequent, posing a diagnostic challenge for malignancy [9].

Histology remains a critical component of the diagnosis of AIP type 1. Extensive lymphoplasmacytic infiltration rich in plasma cells, fibrosis at least focally arranged in a storiform pattern, and obliterative phlebitis are the three key histological findings mentioned. Two other features that have been reported in the literature but are neither sensitive nor specific for diagnosis are phlebitis without luminal obliteration and an increase in eosinophils [10]. Immunohistochemistry shows a predominance of IgG4-positive plasma cells. A number of more than 50 IgG4-positive plasma cells per high-power field (HPF) has been described in the literature and appears to be specific, as it has never been described in any other pancreatic pathology [11-15]. It should be emphasised that histological and anatomopathological evaluations are rarely available prior to surgical resection, as most surgical teams do not perform a biopsy in the case of pseudotumours suggesting malignancy. This may account for the 5-10% incorrect surgical procedures reported in the literature [16,17].

Given the lack of specificity of the clinical and even radiological features of AIP in particular and IgG4 disease in general, the diagnosis of IgG4-related AIP can sometimes be a real challenge, requiring the collection of a number of arguments. Many guidelines have been proposed for the diagnosis of igG4-related disease; the most commonly used criteria in the literature are those proposed by a Japanese consensus, called "Comprehensive Diagnostic Criteria" or CDC [18]. These are general criteria that are based on a combination of clinical, biological, and histological criteria and are used to categorize patients as having "possible" IgG4-related disease (in the presence of clinical and biological criteria), "probable" (in the presence of clinical and histological criteria) or "definite" (in the presence of the three criteria) [18]. A lack of biopsy samples meant that these criteria had relatively low sensitivity in individuals with IgG4-related AIP [19]. The identification of additional diagnostic criteria, such as the 2019 ACR/EULAR IgG4-RD classification [20], has therefore proven crucial in addressing the challenges posed by ambiguous cases. One of the defining characteristics of this diagnostic criteria set is that a patient can be reliably identified as having IgG4-RD even in the absence of a biopsy or an elevated serum IgG4 level in a significant number of cases [20]. In our case, the diagnosis of IgG4-related AIP was maintained based on histological and immunohistochemical findings.

In accordance with the aforementioned criteria, the diagnosis of AIP can be made without the need for invasive methods, provided that the presence of adequate radiological imaging and raised serum IgG4 levels can be demonstrated. Nevertheless, certain cases, such as those with a pseudotumor phenotype that initially suggests pancreatic cancer, may prove challenging to diagnose. In such cases, it is imperative to perform a pancreatic biopsy in order to prevent the implementation of an unwarranted surgical intervention.

In contrast to pancreatic adenocarcinoma, where surgical intervention is frequently indicated, simple corticosteroid therapy is typically sufficient to treat AIP. Nevertheless, immunotherapy may be necessary in the event of a relapse or resistance.

## Conclusions

The diagnosis of AIP is based on a combination of clinical, biological, radiological, and anatomopathological evidence. Effective communication between the clinician, radiologist, and pathologist is therefore essential to ensure a correct diagnosis.

It is critical to distinguish pancreatic cancer from IgG4-related AIP, as the two conditions have completely different prognoses and require different treatments. A misdiagnosis as a malignancy can lead to inappropriate surgical interventions. It is therefore imperative that a biopsy be conducted prior to any invasive treatment, including surgery, given that the associated complications merit due consideration.
